# Evaluating screening and intervention for poverty in family practice settings in Saskatchewan in the context of the COVID-19 pandemic

**DOI:** 10.1017/S1463423625100716

**Published:** 2026-01-08

**Authors:** Loreanne Manalac, Madeline Collins, Olivia Robertson, Adam Clay, Rejina Kamrul

**Affiliations:** Department of Family Medicine, College of Medicine, University of Saskatchewan, Regina, Saskatchewan, Canada

**Keywords:** family, nurse practitioners, physicians, poverty, primary health care, Saskatchewan, social determinants of health

## Abstract

**Objective:**

Poverty is a risk factor for poor health. We sought to determine the practices, barriers, knowledge and comfort with poverty screening and intervention amongst family physicians (FPs), family medicine residents (FMRs) and family nurse practitioners (NPs) in Saskatchewan, Canada during the COVID-19 pandemic.

**Methods:**

A survey was distributed by email and newsletters to FPs, FMRs and NPs in Saskatchewan during 2022.

**Results:**

Eighty-three FPs, 35 FMRs and 25 NPs responded. Time, patient factors, practitioner knowledge and availability of community resources/services were reported barriers. Comfort discussing government benefits with patients was low, with slight differences amongst provider groups (*p* =.042). Thirty-one (40.3%) FPs, 7 (20.6%) FMRs and 17 (68.0%) NPs had referred a patient to a government benefit. Eight (6%) respondents used the Poverty Screening Tool.

**Discussion:**

Further research and training is needed to integrate poverty screening and intervention into primary care, given practitioners’ role as healthcare’s initial point of contact.

Socioeconomic status and income are the major drivers in the overall health outcome of patients, and one of the most important social determinants of health. Studies worldwide have shown that persons living in poverty and income insecurity face a higher rate of morbidity and mortality (Marmot, 2005; Stein Berman *et al.*, 2018). Compared to those in the highest income group, Canadians in lowest-income group have higher rates of arthritis (+7.3%), hypertension (+6.1%), diabetes (+5.1 %), chronic obstructive pulmonary disease (+4.8%), heart disease (+3.8%), history of cancer (+2.2%) and stroke (+1.6%) (Statistics Canada, 2023). Those experiencing income inequality have also been shown to have increased risk of mental illness, with a 58% higher risk of depression than the Canadian average (Smith *et al.*, 2007). Accidents and traumas have also been related to poverty (Oliver and Kohen, 2009). Income inequality has shown to be associated with the premature deaths of 40,000 Canadians per year (Tjepkema *et al.*, 2013).

Despite living in a high-income country, millions of Canadians continue to live in poverty. Canada has adopted 17 Sustainable Development Goals to end poverty and aims to reduce the rate of poverty by half by 2030 (Dionne and Raymond-Brousseau, 2018). Prior to the COVID-19 pandemic, poverty and low income were on the decline in Canada (Statistics Canada, 2022), a trend that continued during the first year of the COVID-19 pandemic. However, the rates of poverty increase from a low of 6.4% in 2020 to 9.9% in 2022 after COVID-19 specific income support programs ended (Dionne and Raymond-Brousseau, 2025). Pandemics such as COVID-19 can emphasize social and health inequalities (Clouston *et al.*, 2021). Before and after the COVID-19 pandemic, economic instability and financial strain have impacted the people living in Canada and worldwide (Dionne and Raymond-Brousseau, 2025). Apart from the direct health effects of the pandemic and social isolation, many individuals faced job loss due to lockdowns which exacerbated the impact of social determinants of health (Garba *et al.*, 2022).

With the overwhelming body of evidence linking poverty with poor health outcomes, family medicine organizations in the United States of America and Canada have called to address poverty as a health issue, and to identify tangible next steps to address poverty (American Academy of Family Physicians, 2015; College of Family Physicians of Canada, 2015). Screening for poverty during healthcare visits has been suggested (American Academy of Family Physicians, 2015; College of Family Physicians of Canada, 2015). Canada is a leader in advocating for such screening through the use of the Poverty Screening Tool (van Buuren *et al.*, 2021), with its use endorsed by the College of Family Physicians of Canada. The tool recommends all patients be screened with the question, ‘Do you (ever) have difficult making ends meet at the end of the month?’. This question has a sensitivity of 98% and a specificity of 40% for identifying those living under the poverty line (Brcic *et al.*, 2011). Filing tax forms can allow access to government benefits and a tax refund in Canada. Thus, the tool’s next question is ‘Have you filled out and sent your tax forms?’ Primary care–based social interventions offer an important means to mitigate threats to individual and community health posed by adverse social conditions (Bloch and Rozmovits, 2021). The Poverty Screening Tool then provides interventions to address poverty, including referral of patients to appropriate government and community resources. The Government of Canada rolled out several COVID-specific benefits to address financial needs during the pandemic (Government of Canada, 2022) that were not specifically addressed by the Poverty Screening Tool. Due to its lower specificity, the screening question may identify higher-income individuals with financial difficulties. However, many of the suggested government programs are eligible to individuals with higher income and would still provide benefit.

Despite the theoretical benefits of screening for poverty, it is not a routine part of family practice (Purkey *et al.*, 2019). An exploratory study investigating the ‘real world’ implementation of the poverty screening tool in a primary and paediatric care setting found that despite high motivation to screen for poverty, only 9% of patients were screened (Purkey *et al.*, 2019). Poverty may not always be recognizable in patients, but in Ontario, Canada it was found that 20% of families or 1.57 million people are living below the poverty line, with higher risk in new immigrants, women, indigenous peoples, LGBTQ+, and children (Bloch *et al.*, 2021). When encouraged to use the poverty screening tool in a clinic servicing low-income individuals including a large number of refugees and newcomers, physicians used the tool in approximately 20% of their patient encounters, with an average of less than five patients for each physician during the one-month study period (Pinto *et al.*, 2019). Twenty-five percent of at-risk individuals were screening after adding prompts to electronic medical record (EMR) charts to screen patients living in more deprived areas (Wintemute *et al.*, 2021). Health care providers identified lack of time, physician discomfort, and lack of expertise as barriers to routine poverty screening (Pinto *et al.*, 2019; Wintemute *et al.*, 2021).

Canadian studies investigating poverty screening have mainly focused on physicians in a small number of clinics in the provinces of Ontario and British Columbia. Primary care in Canada is publicly funded with the delivery shared between provincial governments and the federal government. As such, provincial data are required to inform policy and training needs. There is a lack of evidence on the use of poverty screening amongst primary care providers in other Canadian provinces, including Saskatchewan. This is despite Saskatchewan having high rates of poverty than the Canadian population from 2018 to 2023 (Statistics Canada, 2025). Moreover, there is a lack of information on the screening behaviours of nurse practitioners (NPs), and the impact of the COVID-19 pandemic on poverty screening. Our study investigated the practices, barriers, comfort-levels and knowledge of poverty screening and intervention amongst family physicians (FPs), family medicine residents (FMRs), and family NPs in Saskatchewan during the COVID-19 pandemic.

## Methods

A cross-sectional survey was developed and distributed to FPs, family medicine residents and NPs in Saskatchewan in the winter of 2022. Family physicians, family medicine residents and NPs currently practising in Saskatchewan were included. Exclusion criteria involved specialist physicians (who do not provide primary care in Canada) and medical students. This project was reviewed and approved by the University of Saskatchewan’s Behavioural Research Ethics Board (ID 3035).

A multi-modal recruitment strategy was used to maximize the networking potential of the study team and Saskatchewan’s professional networks to distribute survey invitations (McRobert *et al.*, 2018). Survey invitations were distributed by the email or electronic newsletters of relevant professional societies/groups (i.e. physicians through Saskatchewan Medical Association and Staff Practitioner Affairs division of the Saskatchewan Health Authority; nurses through Saskatchewan Registered Nurses Association and Saskatchewan Association of Nurse Practitioners). Family medicine residents were invited by email through the University of Saskatchewan’s Department of Academic Family Medicine. A chance to win one of four $50 Amazon gift cards was offered as an incentive. Data collection was conducted through SurveyMonkey. Questions were developed based on the Poverty Screening Tool (Bloch, 2016), previously identified barriers and attitudes from other studies (Pinto *et al.*, 2019; Purkey *et al.*, 2019; Wintemute *et al.*, 2021), and financial resources identified on the Government of Canada website (Government of Canada, 2022). The survey questions (reflected in the results tables) were composed of multiple choice and Likert items. These questions were divided in into sections including demographics, barriers to poverty screening, level of comfort in discussing specific benefits, and practices/knowledge/attitudes around poverty screening.

Continuous variables and Likert items were described as medians with interquartile ranges, whereas categorical variables were described as frequency and percentages. Intergroup comparisons were performed using a Kruskal–Wallis test (Likert items), Likelihood ratio or Pearson Chi-squared test, as appropriate. As we did not have a primary outcome of interest and invited all eligible participants, we did not calculate *a priori* sample size. The significant level was set to an alpha of 0.05. All analysis was performed using IBM SPSS version 28.

## Results

### Demographics

One-hundred forty-three surveys were analysed after excluding 27 responses for providing only demographic information, prior completion of the survey, not practising in Saskatchewan, or a specialty practice. Thirty-five FMRs (response rate = 32%), 25 NPs and 83 FPs participated in the survey. We cannot calculate an accurate response rate for FPs and NPs because we do not how many individuals were reached by emails and newsletters. However, there were 236 NPs (Canadian Institute for Health Information, 2024a) and 1271 FPs (Canadian Institute for Health Information, 2024b) in Saskatchewan in 2022. Thus, 10.6% of NPs and 6.7% of FPs in the province completed the survey. Demographic information of respondents is presented in Table 1.

**Table 1.** Demographics of respondents to survey of Saskatchewan primary care providers in winter 2022. Values represent count (percent)

		Family medicine physician (*n* = 83)	Family medicine resident (*n* = 35)	Nurse practitioner (*n* = 25)
Gender	Male	26 (31.3%)	8 (22.9%)	0 (0%)
	Female	56 (67.5%)	23 (65.7%)	25 (100%)
	Prefer not to disclose/self-described	1 (1.2%)	4 (11.4%)	0 (0%)
Years of practice	0–5	17 (20.5%)	–	7 (28.0%)
	6–10	29 (34.9%)	–	4 (16.0%)
	11–20	13 (15.7%)	–	11 (44%)
	> 20	24 (28.9%)	–	3 (12.0%)
Type of practice*	Contract	36 (43.4)	–	1 (4%)
	Fee for service	51 (61.4%)	–	0 (0%)
	Employee	4 (4.8%)	–	25 (100%)
Location of practice*	Urban (>100,000)	44 (53.0%)	20 (57.1%)	8 (32.0%)
	Small Urban (10,000–100,000)	18 (21.7%)	12 (34.3%)	1 (4%)
	Rural or remote (<10,000)	23 (27.7%)	7 (20.0%)	16 (64%)

* Respondents could select more than one response to reflect mixed practices.

### Barriers to poverty screening

Results indicated barriers to screening from poverty included time during visits to screen, patients’ willingness to discuss their finances, priority of other health concerns, availability of resources, and health care provider awareness of resources, referral process, and ability to provide information about resources in the community (see Table 2). There was no difference in the average agreement with barriers between healthcare providers (*p* = .071).

**Table 2.** Saskatchewan-based family physician, family medicine resident and nurse practitioner level of agreement with different barriers to poverty screening and intervention. Values represent median (IQR) of responses*

	Family medicine physician (*n* = 83)	Family medicine resident (*n* = 35)	Nurse practitioner (*n* = 25)
Do you agree the following are barriers to screening and intervening for poverty in your practice?			
Time during visits to screen for poverty.	4 (3–4)	4 (4–5)	3 (2–4)
Time during visits to provide information, referral to services addressing income, tax services and employment in my community.	4 (4–5)	4 (4–5)	4 (3–4)
Patients’ willingness to discussing their financial situations/if they are struggling financially.	4 (2–4)	4 (3–4)	3 (2–4)
Patients prioritize other health priorities over their financial needs.	4 (2–4)	4 (3–4)	3 (2–4)
Availability of community resources and services that address income, tax services and employment.	4 (4–5)	4 (3–5)	4 (4–5)
My awareness of the resources and services addressing income, tax services and employment in my community.	4 (4–5)	5 (4–5)	4 (2–5)
My knowledge of the referral process to community services that address income, tax services and employment in my community.	4 (4–5)	4 (4–5)	4 (2–5)
My ability to provide information to patients regarding community services that address income, tax services and employment in my community.	4 (3–5)	4 (4–5)	4 (3–5)
Average agreement with barriers	4 (3.6–4.3)	4.0 (3.6–4.4)	3.6 (2.8–4.1)

* The response options were 1 (strongly disagree), 2 (disagree), 3 (neutral), 4 (agree) and 5 (strongly agree).

### Level of comfort

Respondents reported lack of comfort in explaining government benefits to patients. Nurse practitioners were more comfortable than FMRs (*p* = .042). There was no different in comfort reported between FMRs, FPs or NPs (*p* = .066). Results are reported in Table 3.

**Table 3.** Saskatchewan-based family physician, family medicine resident and nurse practitioner agreement with statements that they were comfortable discussing government benefits with patients. Values represent median (IQR) of responses*

		Family medicine physician (*n* = 77)	Family medicine resident (*n* = 34)	Nurse practitioner (*n* = 25)
I am comfortable educating my patients regarding the following income security benefits:	Old Age Security	2 (1–4)	2 (2–2)	2 (2–4)
	Guaranteed Income Supplement	2 (1–3)	2 (2–2)	2 (2–3)
	Canada Child Benefit	2 (1–4)	2 (2–4)	4 (2–4)
	Non-insured Health Benefits	3 (2–4)	2 (2–4)	4 (2–4)
	Average Comfort with pre–existing benefits	2.5 (1.8–3.5)	2.3 (2.0–3.0)	3.0 (2.5–3.5)
I am comfortable discussing the following COVID-related benefits with my patients:	Canada Recovery Benefit	2 (1–2)	2 (2–3)	2 (2–3)
	Canada Recovery Caregiving Benefit	2 (1–2)	2 (2–2)	2 (2–2)
	Canada Recovery Sickness Benefit	2 (1–2)	2 (1–2)	2 (2–2)
	Canada Emergency Wage Subsidy	2 (1–2)	2 (1–2)	2 (2–3)
	Canada Emergency Rent Subsidy	2 (1–2)	2 (1–2)	2 (2–2)
	Mortgage Payment deferral	2 (1–2)	2 (1–2)	2 (2–4)
	Saskatchewan’s Temporary Wage Supplement	2 (1–2)	2 (1–2)	2 (2–2)
	Average comfort with COVID Benefits	2 (1–2.4)	2 (1.7–2.0)	2.14 (2–2.6)

* The response options were 1 (strongly disagree), 2 (disagree), 3 (neutral), 4 (agree) and 5 (strongly agree).

### Poverty screening integration, knowledge, and attitudes

Respondents were generally not using the Poverty Screening Tool. Nurse practitioners referred more than family medicine physicians and residents to tax clinics/income support (*p* = 0.001) and to government benefits (*p* < 0.001). Respondents generally agreed that additional training in poverty screening should be provided and disagreed that they currently have sufficient education on the topic. Information on screening, knowledge and attitudes towards poverty screening is provided in Table 4.

**Table 4.** Saskatchewan-based family physician, family medicine resident and nurse practitioner responses to questions regarding knowledge, practice and attitudes. Values represent count (%) for yes/no items or median (IQR) for Likert items*

		Family medicine physician (*n* = 77)	Family medicine resident (*n* = 34)	Nurse practitioner (*n* = 25)	*P*-value
Practice	I routinely screen for poverty with patients in my practice at least yearly.	3 (2–4)	2.5 (2–3)	4 (2–5)	.263
	In the past year, I have referred patients to the community tax clinic or income support services in my community if they are available**	1 (1–3)	1 (1–1)	2 (1–4)	.001
	I have used the Poverty Screening Tool in practice (yes/no)	3 (3.9%)	2 (5.9%)	3 (12.0%)	.430
	I have referred patients to any of the government income security benefits.	31 (40.3%)	7 (20.6%)	17 (68.0%)	<.001
	I am screening more often for poverty because of the COVID-19 pandemic.	3 (2–4)	2 (2–3)	2 (2–3)	.554
Knowledge	I am familiar with the Centre for Effective Practice’s Poverty Screening Tool	8 (10.3%) *n* = 78	3 (8.8%)	4 (16.0%)	.740
	I feel confident referring patients to community services to support their financial needs (i.e. food insecurity, tax clinics).	2 (2–3)	2 (2–3)	3 (2–4)	.256
	I recognize which patients might benefit from or be eligible for employment insurance.	3 (2–4)	3 (2–3)	3 (2–4)	.083
	I am familiar with the current COVID-19 benefits and services available through the Government of Canada.	2 (2–3)	2 (2–3)	2 (2–3)	.379
	I know where I can direct patients to receive more information and applications for COVID-19 benefits (yes/no)	27 (36.0%) *n* = 75	12 (36.4%), *n* = 33	11 (44%)	.766
Attitudes	I feel like I have a role in intervening in situations of poverty for my patients.	4 (3–4)	4 (3–4)	4 (4–4)	.680
	I have received enough training, either in medical school, residency, nurse practitioner school or in continuing medical education to screen and intervene for poverty.	2 (1–2)	2 (2–3)	2 (2–3)	.289
	Supplemental training or resources should be provided to physicians/ family medicine residents/nurse practitioners to better equip them to screen and intervene for poverty.	4 (4–5)	4 (4–5)	5 (4–5)	.120
	I feel encouraged to screen for poverty following the COVID-19 pandemic.	3 (2–4)	3 (2–4)	3 (2–4)	.895

* The response options were 1 (strongly disagree), 2 (disagree), 3 (neutral), 4 (agree) and 5 (strongly agree).

** The response options were 1 (Never), 2 (Very Rarely), 3 (Rarely), 4 (Occasionally), 5 (Frequently).

## Discussion

This study revealed poverty screening is not routinely practised by many FPs, FMRs and family NPs in Saskatchewan. Comfort discussing government benefits with patients was generally low, with slight differences amongst provider groups. These results suggest that NPs, in addition to physicians, could improve their poverty screening practices.

Barriers faced by respondents include time constraints, other priorities during visits, knowledge gaps in the referral process to community resources and lack of comfort in screening patients. These barriers are similar to those previously reported (Pinto *et al.*, 2019; Purkey *et al.*, 2019). Despite the COVID-19 pandemic, respondents did not change their poverty screening practices, which is contradictory to patients’ current needs given the financial hardships of the pandemic. Respondents also reported less comfort discussing pandemic specific benefits (e.g., Canada Recovery Benefit) than pre-existing benefits (e.g., Old Age Security). Routine primary health care was impacted by the COVID-19 pandemic (Taylor *et al.*, 2022). It is possible the low levels of screening in the current study may be due to priority of acute health care concerns during the pandemic or a shift from in-person to virtual visits.

Primary care providers in Saskatchewan believed it is important to screen for poverty but did not feel that they have received adequate training or resources. This is consistent with findings amongst primary care physicians in other work (Purkey *et al.*, 2019). Only a few respondents were familiar with the Poverty Screening Tool and had used it in their practice. Respondents identified gaps in their knowledge of the practical components of the Poverty Screening Tool. These gaps involve referral, education around government benefits and programs that could stimulate improvement in the overall health outcome of the patient. While the questions include in the tool do not take much time, follow up conversations are time consuming with one study indicating providers typically booked 30-minute appointments (Pinto *et al.*, 2019). Primary care providers have a unique opportunity and are well positioned to be the first point of contact of help for many patients (Pinto *et al.*, 2019; Starfield *et al.*, 2005). They have an opportunity to see patients holistically and establish rapport with continued visits.

Learning opportunities may help address the gap in primary care providers’ knowledge and confidence related to poverty screening and intervention. Learning modules centred on incorporating poverty screening, or other social determinants of health, equity and patient advocacy could be added into the undergraduate and postgraduate medical and nursing curriculum. For those currently practising, Continuing Medical Education (CME) can provide updates on government benefits and resources available on a provincial and federal level, as well as those delivered by the provincial health authority. Notably, education alone is not effective in optimizing clinical care by health professionals (Clyman *et al.*, 2007). Thus, strategies aimed at reducing barriers to screening and further identifying facilitators in different practice models (i.e., fee for service vs salary, rural vs urban) are needed. For example, several organizations have called for the integration of public health and primary care to address social determinates of health (Tong *et al.*, 2018). Saskatchewan is currently expanding access to team-based care, that may include physicians, nurses, pharmacists, physiotherapists, dietitians, mental health counsellors, social workers, and other professionals (Saskatchewan Health Authority, 2021; Government of Saskatchewan, 2025). This may be an opportunity to increase the rate of poverty screening, as physicians have indicated that allied health care staff and social works may be most appropriate for using Poverty Screening Tool (Pinto *et al.*, 2019). Collaboration between primary care providers and other groups may help develop pathways that facilitate easier referrals to tax clinics or community resources. Collaboration may also help connect individuals without a primary care provider (i.e., without a family doctor) with appropriate resources.

Other strategies to reduce screening barriers could include addition of EMR prompts during periodic health exams or information sheets available in the office for patient use (Pinto, 2019). Family physicians and primary care NPs have a unique position as the healthcare first point of contact and likely have a deeper knowledge of the patient’s medical and social history than other providers. Their intervention may pose a significant impact on the patient’s health outcome, but this has yet to be determined.

This study had several limitations. Due to the nature of our recruitment methods (newsletters and emails) it is unknown how many providers were reached, and we could not calculate an accurate response rate for FPs and NPs. Thus, the results may be impacted by non-response bias and the small sample size impacts generalizability. Physician surveys may be less prone to non-response bias than surveys of other groups due to similarities in accreditation standards in medical training (Kellerman and Herold, 2001). Our analysis was focused on overall trends and differences in responses between three main categories of primary care providers in Saskatchewan. We did not correct for multiple comparison which increases the likelihood of type I error, as this was an exploratory analysis (Althouse, 2016; Streiner and Norman, 2011). Future analysis could involve exploring the differences in responses between other demographic categories such as rural versus urban physicians or practice payment model.

## Conclusions

Primary health care practitioners recognize the importance of screening for poverty amongst their patients although most do not routinely screen in their practice, despite the economic pressures caused by the COVID-19 pandemic. Barriers to screening include time constraints, lack of training and a decreased level of comfortability and knowledge in screening for and addressing poverty. The findings in this study demonstrate a need for further research and training to integrate poverty screening and intervention in primary care with an aim to offer more holistic primary care by incorporating the social needs of the patient.

## Data Availability

The data that support the findings of this study are available on request from the corresponding author. The data are not publicly available due to privacy or ethical restrictions.
